# Pathogenesis and transmission of human seasonal and swine-origin A(H1) influenza viruses in the ferret model

**DOI:** 10.1080/22221751.2022.2076615

**Published:** 2022-06-01

**Authors:** Joanna A. Pulit-Penaloza, Nicole Brock, Joyce Jones, Jessica A. Belser, Yunho Jang, Xiangjie Sun, Sharmi Thor, Claudia Pappas, Natosha Zanders, Terrence M. Tumpey, C. Todd Davis, Taronna R. Maines

**Affiliations:** Centers for Disease Control and Prevention, Influenza Division, National Center for Immunization and Respiratory Diseases, Atlanta, GA, USA

**Keywords:** Influenza, ferret, transmission, pathogenesis, variant

## Abstract

Influenza A viruses (IAVs) in the swine reservoir constantly evolve, resulting in expanding genetic and antigenic diversity of strains that occasionally cause infections in humans and pose a threat of emerging as a strain capable of human-to-human transmission. For these reasons, there is an ongoing need for surveillance and characterization of newly emerging strains to aid pandemic preparedness efforts, particularly for the selection of candidate vaccine viruses and conducting risk assessments. Here, we performed a parallel comparison of the pathogenesis and transmission of genetically and antigenically diverse swine-origin A(H1N1) variant (v) and A(H1N2)v, and human seasonal A(H1N1)pdm09 IAVs using the ferret model. Both groups of viruses were capable of replication in the ferret upper respiratory tract; however, variant viruses were more frequently isolated from the lower respiratory tract as compared to the human-adapted viruses. Regardless of virus origin, observed clinical signs of infection differed greatly between strains, with some viruses causing nasal discharge, sneezing and, in some instances, diarrhea in ferrets. The most striking difference between the viruses was the ability to transmit through the air. Human-adapted viruses were capable of airborne transmission between all ferret pairs. In contrast, only one out of the four tested variant viruses was able to transmit via the air as efficiently as the human-adapted viruses. Overall, this work highlights the need for sustained monitoring of emerging swine IAVs to identify strains of concern such as those that are antigenically different from vaccine strains and that possess adaptations required for efficient respiratory droplet transmission in mammals.

## Introduction

Influenza A and B viruses are important human respiratory pathogens capable of causing a substantial public health burden every year. According to the World Health Organization, annual influenza virus epidemics result in approximately 3–5 million cases of severe illness, and up to 650,000 respiratory deaths worldwide [1]. While influenza B viruses are limited to humans, the ecology of IAVs is complex and involves a broad range of avian and mammalian species. As such, these viruses have the potential of crossing the species barrier and causing a pandemic [2]. IAVs, which are divided into subtypes based on the type of surface glycoproteins [hemagglutinin (HA) and neuraminidase (NA)], have evolved into several host-specific lineages. Aquatic birds represent the main natural reservoir with the greatest IAV diversity; however, mammalian species, including but not limited to humans, pigs, horses, and dogs also maintain genetically and antigenically distinct virus lineages [3, 4]. Several influenza IAV pandemics have been documented in the past [5]. The most recent pandemic occurred in 2009 and was caused by a swine-origin, quadruple reassortant A(H1N1) virus [referred to as A(H1N1)pdm09], which subsequently replaced the human seasonal A(H1N1) virus strain and continues to circulate in the human population [6]. Because sporadic human infections with swine-origin influenza viruses, referred to as variant viruses [including A(H1N1)v, A(H1N2)v, and A(H3N2)v] are reported annually, there is great interest in evaluating the pandemic potential of these viruses. Surveillance and genetic analysis efforts revealed that pigs can occasionally be infected with avian IAV strains but also that humans transmit IAVs to pigs more frequently than pigs do to humans [7]. Due to multi-directional transmission of influenza viruses, reassortment, and accumulation of mutations, pigs have become a reservoir of very diverse, rapidly evolving virus populations to which humans frequently have limited or no immunity [8, 9]. As these viruses typically lack adaptations necessary for efficient human-to-human transmission, occasional zoonotic infections present an opportunity for the virus to adapt to the new host [10]. For these reasons, continuous surveillance of swine IAVs, including characterization of genetic and antigenic diversity, as well as assessments of pathogenicity and transmissibility using mammalian models are critical activities for influenza risk assessments and for the selection and generation of candidate vaccine viruses (CVVs) for pandemic preparedness.

In this study, we used the ferret model to assess the pathogenesis and transmission capability of four genetically and antigenically diverse swine A(H1) IAVs isolated from humans between 2017 and 2020. Three contemporary A(H1N1)pdm09 viruses were included in the analysis for comparison. The findings of this study provide insight into the evolution of swine IAV, supporting that these viruses can emerge into genetically and antigenically distinct strains that in some cases are capable of transmission via the air in the ferret model at comparable levels of efficiency as seen for human-adapted viruses.

## Materials and methods

*Viruses*. Stocks of A/Alberta/1/2020 A(H1N2)v, A/Ohio/24/2017 A(H1N2)v, A/California/62/2018 A(H1N2)v, A/Michigan/288/2019 A(H1N1)v, A/Michigan/45/2015 A(H1N1)pdm09, A/Idaho/7/2018 A(H1N1)pdm09, and A/Nebraska/14/2019 A(H1N1)pdm09 viruses were propagated in Madin-Darby Canine Kidney (MDCK) cells at 37°C for 48 hrs. Viral titers were determined using a standard plaque assay in MDCK cells as described previously [11]. Each stock virus was sequenced and tested for exclusivity to rule out the presence of other subtypes of influenza virus. All work was conducted in a BSL2-enhanced or higher laboratory.

*Phylogenetic analysis*. A phylogenetic tree was built for the influenza virus HA genome segment obtained from GISAID (http://platform.gisaid.org). The sequences for each strain were aligned using the ClustalW application and Muscle algorithm [12]. Neighbour joining phylogenetic trees were built using MEGA7.0 software with 1000 bootstraps and the Jukes-Cantor Model of evolution with uniform rates (www.megasoftware.net) [13]. Secondary analysis was performed using MAFFT and IQ-tree confirming the locations and fidelity of the represented NJ phylogenetic tree topology.

*Antigenic analysis*. Ferret immune sera were harvested 14 days post intranasal inoculation with 6 log_10_ 50% egg infectious dose (EID_50_) of virus. Two-way hemagglutination inhibition (HI) assay was used to assess cross-reactivity between different virus strains and pooled human sera (with human subject research approval [14]) as previously described [15]. Briefly, each serum sample was treated at a 1:4 dilution with receptor-destroying enzyme (RDE, Denka Seiken) for 18 h at 37°C. Physiological saline was added to reach a 1:10 final dilution, followed by adsorption with turkey red blood cells. All antigens were standardized to 8 HAU/50 µl prior to 30 min incubation at room temperature with 2-fold serial dilutions of antiserum. Turkey red blood cells (0.5%) were used to evaluate HI.

*Ferret experiments*. Animal research was conducted under the guidance of the Centers for Disease Control and Prevention's Institutional Animal Care and Use Committee in an Association for Assessment and Accreditation of Laboratory Animal Care International-accredited animal facility. Serologically negative for currently circulating influenza A and B viruses, male Fitch ferrets (Triple F Farms, Sayre, PA) (6-13 months of age) were housed in Duo-Flo Bioclean mobile units (Lab Products Incorporated, Seaford, DE) during experimentation. For each transmission experiment, groups of 3–6 ferrets per virus were anesthetized via intramuscular injection of a ketamine cocktail (20 mg/kg Ketamine, 0.05 mg/kg Atropine, 2 mg/kg Xylazine) in the hamstring and inoculated intranasally with 6 log_10_ PFU diluted in 1 ml of virus diluted in PBS. The next day the Direct Contact Transmission model (DCT) was established by placing a naïve ferret in each cage housing an inoculated ferret (3 ferret pairs per virus tested) and the Respiratory Droplet Transmission model (RDT) was established by placing naïve and inoculated ferrets in adjacent cages separated by a perforated wall (3 ferret pairs per virus tested) [16]. Each ferret was observed daily for clinical signs of infection. Nasal washes were collected every two days for two weeks post inoculation (p.i.) and post contact (p.c.) for determination of virus shedding; briefly, following anesthesia 1 ml volume of PBS was introduced into the ferret nasal passages to induce sneezing and the aspirate was collected in a sterile Petri dish. Three additional ferrets per virus were inoculated as described above and then euthanized on day 3 p.i. for the assessment of systemic spread of virus [17]. Any animal that exhibited ≥25% weight loss or severe illness was humanely euthanized. All ferret samples were analyzed for viral titers using standard plaque assay in MDCK cells [11].

## Results

**Figure 1.**
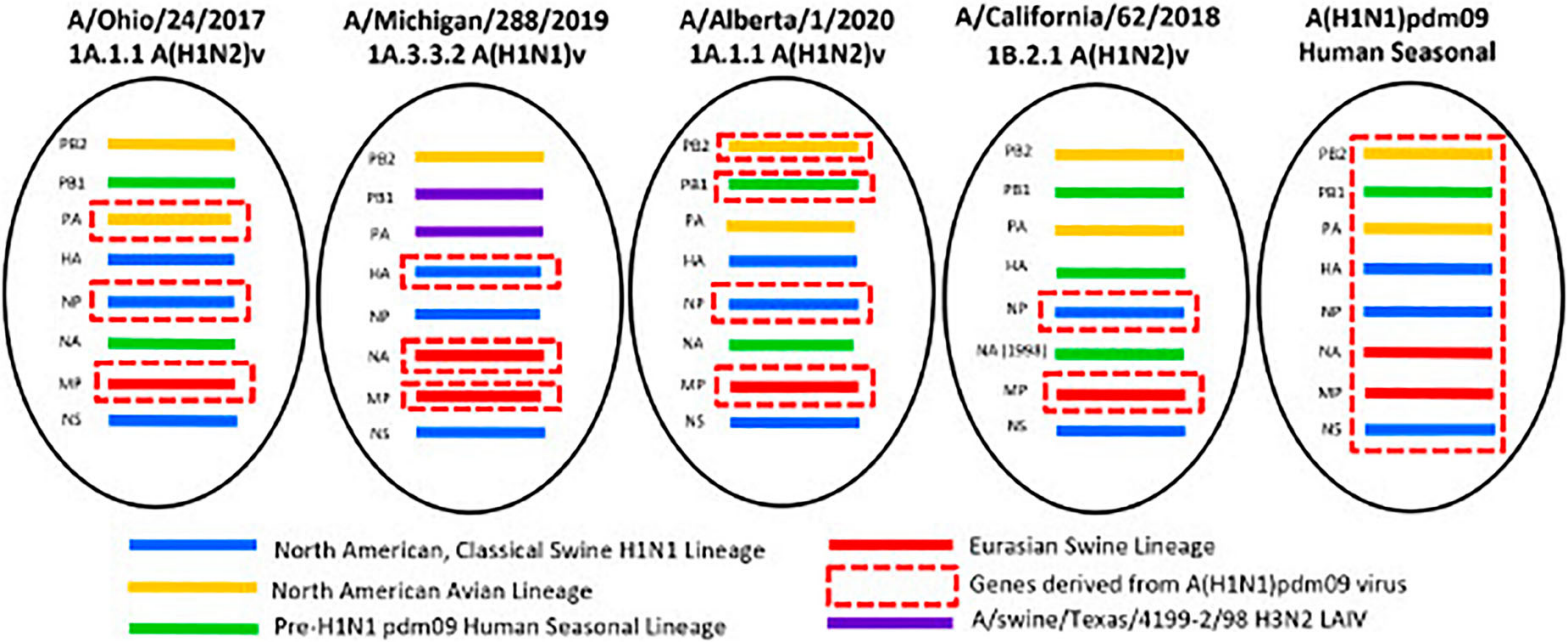
Genome constellation identified in A(H1)v viruses in North America. Genes derived from the following lineages: blue – North American Classical Swine A(H1N1); yellow – North American Avian lineage; green – pre-A(H1N1)pdm09 human seasonal virus; red – Eurasian swine lineage; dashed red box – A(H1N1)pdm09 virus genes; purple – genes derived from A/swine/Texas/4199-2/98 A(H3N2) live attenuated influenza vaccine virus.

*Phylogenetic analysis*. Phylogenetic analysis was performed using the sequences of the mature HA proteins with the signal peptide removed. Four genetically distinct A(H1)v viruses associated with recent human infections were examined. The A(H1N2)v virus detected in Alberta, Canada in 2020 (A/Alberta/1/2020, referred to as Alberta/1) clustered with alpha (1A.1.1) lineage swine IAVs circulating in Canada and the USA (Supplementary Figure 1A). Alberta/1 and A/Ohio/24/2017 (OH/24), also an alpha (1A.1.1) lineage A(H1N2)v virus, evolved from classical swine IAVs that have circulated in North America since they were first detected in the 1930s [18]. Genotypic analysis revealed that both viruses had genes derived from A(H1N1)pdm09 virus; PA, NP, and MP in case of OH/24 and PB2, PB1, NP, and MP in case of Alberta/1 (Figure 1). A/Michigan/288/2019 (MI/288) A(H1N1)v virus was previously phylogenetically classified as alpha lineage (1A.3.3.2) [15], while the HA and NA of this virus clustered with contemporary human A(H1N1)pdm09 viruses (clade 6B.1A) and swine IAVs from the USA; all of the internal protein coding vRNAs were similar to swine viruses circulating in the USA (Figure 1). The PB1 and PA genes clustered with those of the A/swine/Texas/4199-2/1998 virus used in a commercial, live-attenuated swine vaccine in the USA. In contrast to 1A.1.1 lineage viruses, lineage 1B.2.1 viruses circulating in USA swine, represented in this study by A/California/62/2018 (CA/62) A(H1N2)v, are remnants of pre-2009 human seasonal A(H1N1) viruses that have been maintained in pigs since these viruses were displaced by A(H1N1)pdm09 viruses in humans after the beginning of the 2009 pandemic (Supplementary Figure 1B). Genotypic analysis revealed that the CA/62 virus has pre-A(H1N1)pdm09 human seasonal origin HA, NA, and PB1 and following the pandemic it acquired NP, and MP genes from the A(H1N1)pdm09 virus (Figure 1). Collectively, these analyses support that A(H1)v viruses isolated from humans between 2017 and 2020 possess diverse genetic constellations due to reassortment in pigs.

## Antigenic analysis

Next, we examined antigenic profiles of the swine A(H1)v viruses. Ferret antisera raised to the North American OH/24 and the reverse genetics-derived OH/24-like CVV (IDCDC-RG59) failed to inhibit Alberta/1 in HI tests. Likewise, no cross-reactivity of ferret antisera raised to the recommended seasonal vaccine strain, A/Idaho/7/2018 A(H1N1)pdm09 (ID/7), was observed when tested with this virus. In addition, pooled post-vaccination antisera from children (0-3 years) and adults (19-49 years) also showed little to no cross-reactivity with the Alberta/1 virus (Table 1). In contrast, as reported by Cook et al., when MI/288 was tested by HI it was well inhibited by both ferret antisera raised to an A(H1N1)pdm09 virus (ID/7) and post-vaccination immune sera obtained from vaccinated children and adults [15]. HI testing of pooled, child and adult human post-vaccination sera from persons vaccinated with the 2017–2018 vaccine, as well as ferret sera raised against human seasonal A/Michigan/45/2015 A(H1N1)pdm09 (MI/45) virus, showed no cross-reactivity with the CA/62 virus (Table 2). Similar results were observed for other viruses of either 1B.2.2 (delta 1) or 1B.2.1 (delta 2) lineages. However, ferret antiserum raised to A/Ohio/35/2017 virus, a wild type of strain of the recommended lineage 1B.2.1 (delta 2) CVV, inhibited CA/62 virus. Sera raised to 1B.2.2 (delta 1) strains cross-reacted with the CA/62 virus to varying degrees. Taken together, identification of antigenically distinct IAVs that are capable of human infection underscores the need for continuous surveillance and selection of new CVVs in support of pandemic preparedness.

**Table 1.** Comparative hemagglutination inhibition assay assessment of 1A.1.1 (alpha) lineage A(H1N2)v viruses.

		Ferret Antisera				
REFERENCE ANTIGENS	Subtype (Lineage)	ID/7	OH/24	IDCDC-RG59	Child^a^	Adult^b^
A/Idaho/7/2018	A(H1N1)pdm09	2560	80	40	1280	1280
A/Ohio/24/2017	A(H1N2)v (1A.1.1; alpha)	320	2560	640	NT	20
IDCDC-RG59 (A/OH/24/2017-like)	A(H1N2)v (1A.1.1; alpha)	160	2560	1280	40	20
TEST ANTIGENS						
A/Alberta/1/2020	A(H1N2)v (1A.1.1; alpha)	<10	<10	<10	<10	20

^a^ post-vaccine (2019–2020) immune serum pool from child (0-3 years-old) vaccinees.

^b^ post-vaccine (2019–2020) immune serum pool from adult (19-49 years-old) vaccinees.

NT-not tested.

**Table 2.** Comparative hemagglutination inhibition assay assessment of 1B.2 (delta) lineage A(H1N2)v influenza viruses.

REFERENCE ANTIGENS	Subtype (Lineage)	Ferret Antisera					Child^a^	Adult^b^
		MI/45	MN/45	WI/71	IA/32	OH/35		
A/Michigan/45/2015	A(H1N1)pdm09	5120	10	<10	<10	<10	40	160
A/Minnesota/45/2016	A(H1N2)v (1A.1.1; alpha)	<10	1280	<10	<10	<10	<10	<10
A/Wisconsin/71/2016	A(H1N2)v (1B.2.2; delta 1)	<10	<10	5120	640	<10	<10	<10
A/Iowa/32/2016	A(H1N2)v (1B.2.2; delta 1)	<10	<10	1280	1280	<10	<10	10
A/Ohio/35/2017	A(H1N2)v (1B.2.1; delta 2)	<10	<10	80	20	640	<10	<10
TEST ANTIGENS								
A/California/62/2018	A(H1N2)v (1B.2.1; delta 2)	<10	<10	40	<10	320	<10	<10

^a^ post-vaccine (2017–2018) immune serum pool from child (0–3 years-old) vaccinees.

^b^ post-vaccine (2017–2018) immune serum pool from adult (19–49 years-old) vaccinees.

## Pathogenesis and transmission of A(H1) influenza viruses in the ferret model

Next, we used the ferret model to compare the severity of disease caused by four A(H1)v IAVs (Alberta/1, OH/24, CA/62, and MI/288) and three contemporary seasonal A(H1N1)pdm09 viruses (ID/7, MI/45 and A/Nebraska/14/2019 [NE/14]). Nasal washes were collected every two days p.i. to compare replication capacities and the amount of virus shed from each animal. The peak nasal wash titers ranged between 3.8-7.6 log_10_ PFU/ml for the A(H1)v viruses and between 6.1 and 7.8 log_10_ PFU/ml for the seasonal A(H1N1)pdm09 viruses (Table 3). The Alberta/1 A(H1N2)v virus replicated least efficiently in ferret upper respiratory tract (3.8 log_10_ PFU/ml mean maximum titer in nasal wash samples), inoculated ferrets lost the least amount of weight, and no overt respiratory signs were observed during the time course of infection except for sneezing. In contrary, the ID/7 virus was the most pathogenic in ferrets as evidenced by 20.6% mean maximum loss of pre-inoculation body weight, diarrhea, as well as respiratory signs, including nasal discharge and sneezing. Out of the three inoculated ferrets from this group, one was humanely euthanized on day 9 p.i. due to severe weight loss. Ferrets inoculated with OH/24, CA/62, and MI/288 viruses frequently displayed respiratory signs of infection, transient increase in body temperature, and weight loss (mean maximum 9.0-14.0%), but with diarrhea and mortality were not observed (Table 3). Additional inoculated ferrets (*n* = 3/virus) were sacrificed day 3 p.i. to assess virus dissemination in tissues (Table 4). Low titers of Alberta/1 virus (mean max ≤ 3.3 log_10_ PFU/ml or g) were detected in nasal turbinates and soft palate samples from all tested ferrets, while the virus was found in only 1 trachea and 2 lung samples. ID/7 virus was present in the upper respiratory tract, including the nasal turbinates, soft palate, and trachea, although detection in all three tissues was limited to a single ID/7-infected animal (Table 4). Detection in lungs was observed for all other viruses that were evaluated, albeit not in all ferrets in the Alberta/1 and MI/45 virus group. MI/288 virus mean titer was highest in this tissue (4.8 log_10_ PFU/g). Brain tissue and olfactory bulb were tested for the presence of virus; NE/14 was the only virus detected in both tissues, while MI/288 and ID/7 were detected in the olfactory bulb. Spleen, liver, intestines, kidneys, and blood were also tested but no infectious virus was observed for any of the IAVs included in this study. Overall, while variant A(H1) viruses were detected throughout the ferret respiratory tract, like what was observed with A(H1N1)pdm09 viruses isolated in 2009 [19–22], contemporary human-seasonal viruses were more often restricted to the upper respiratory tract. Although uncommon, lethal phenotypes can be observed with either A(H1)v or A(H1N1)pdm09 viruses as shown here and previously [9, 22].

**Table 3.** Summary results of pathogenesis and transmission of A(H1) viruses in the ferret model.

										Transmission ^f^	
Virus	Name in the study	Subtype	NW titer ^a^	Weight loss (%) ^b^	Temp change (C°) ^c^	Nasal disch. ^d^	Snz. ^d^	Diarrh.^d^	Mortality^e^	DCT	RDT
A/Alberta/1/2020	Alberta/1	A(H1N2)v	3.8 ± 0.5	6.1 (6/6)	1.4	0/6	2/6	0/6	0/6	3/3	1/3
A/Ohio/24/2017	OH/24	A(H1N2)v	5.9 ± 0.9	9.0 (6/6)	1.0	6/6	4/6	0/6	0/6	3/3	1/3
A/California/62/2018	CA/62	A(H1N2)v	6.2 ± 0.6	11.4 (6/6)	1.7	6/6	3/6	0/6	0/6	3/3	1/3
A/Michigan/288/2019	MI/288	A(H1N1)v	7.6 ± 0.4	14.0 (6/6)	1.9	6/6	3/6	0/6	0/6	3/3	3/3
A/Michigan/45/2015	MI/45	A(H1N1)pdm09	6.9 ± 0.4	11.5 (3/3)	1.8	1/3	2/3	0/3	0/3	NT	3/3
A/Idaho/7/2018	ID/7	A(H1N1)pdm09	6.1 ± 0.4	20.6 (3/3)	0.8	3/3	1/3	2/3	1/3 (d9)	NT	3/3
A/Nebraska/14/2019	NE/14	A(H1N1)pdm09	7.8 ± 0.2	11.7 (3/3)	1.0	0/3	1/3	0/3	0/3	NT	3/3

^a^ Average maximum nasal wash (NW) titer expressed as log_10_ PFU/ml ± SD of ferrets inoculated with 6 log_10_ PFU of virus in 1 ml of PBS.

^b^ Average maximum weight loss within 10 days of inoculation. Number of ferrets that displayed weight loss over the total number of animals is in parenthesis.

^c^ Average maximum temperature increase over the baseline (37.7–39.7°C).

^d^ Number of ferrets displaying nasal discharge, sneezing or diarrhea over the total number of animals.

^e^ Number of animals euthanized during the experiment over the total number of animals; day of euthanasia shown in parenthesis.

^f^ DCT: Direct Contact Transmission model; RDT: Respiratory Droplet Transmission model. Number of contact ferrets with detectable virus in nasal washes over the total number of ferrets.

NT not tested.

**Table 4.** Summary results of virus titers in tissues collected on day 3 post inoculation.

Virus	Mean titer (log _10_ PFU/ml or g) ^a^					
	Nasal turbinates	Soft palate	Trachea	Lung	Olfactory bulb	Brain
A/Alberta/1/2020	2.9 ± 0.7 (3/3)	3.3 ± 0.6 (3/3)	2.62 (1/3)	2.6 ± 1.1 (2/3)	ND (0/3)	ND (0/3)
A/Ohio/24/2017	4.4 ± 0.3 (3/3)	4.3 ± 0.1 (3/3)	4.4 ± 1.0 (2/3)	3.8 ± 0.7 (3/3)	ND (0/3)	ND (0/3)
A/California/62/2018	4.9 ± 0.5 (3/3)	5.5 ± 0.7 (3/3)	4.2 ± 0.6 (3/3)	3.1 ± 0.7 (3/3)	ND (0/3)	ND (0/3)
A/Michigan/288/2019	5.4 ± 0.1 (3/3)	5.1 ± 0.5 (3/3)	5.9 ± 0.3 (3/3)	4.8 ± 0.5 (3/3)	2.4 ± 0.6 (3/3)	ND (0/3)
A/Michigan/45/2015	5.4 ± 0.3 (3/3)	3.0 ± 1.1 (3/3)	4.1 ± 0.4 (3/3)	3.2 (1/3)	ND (0/3)	ND (0/3)
A/Idaho/7/2018	4.0 ± 0.6 (3/3)	1.8 (1/3)	1.8 (1/3)	ND ^b^ (0/3)	3.9 (1/3)	ND (0/3)
A/Nebraska/14/2019	5.7 ± 0.1 (3/3)	5.1 ± 0.7 (3/3)	4.7 ± 0.6 (3/3)	4.4 ± 1.5 (3/3)	3.8 ± 2.0 (3/3)	2.5 ± 1.3 (3/3)

^a^ Mean virus titer in tissues collected on day 3 post inoculation ± SD. Nasal turbinate viral titers are presented as log_10_ PFU/ml and trachea and soft palate, trachea, lung, olfactory bulb, and brain titers are presented as log_10_ PFU/g of tissue. Number of ferrets with detectable titers are indicated in parenthesis.

^b^ ND – not detected.

**Figure 2.**
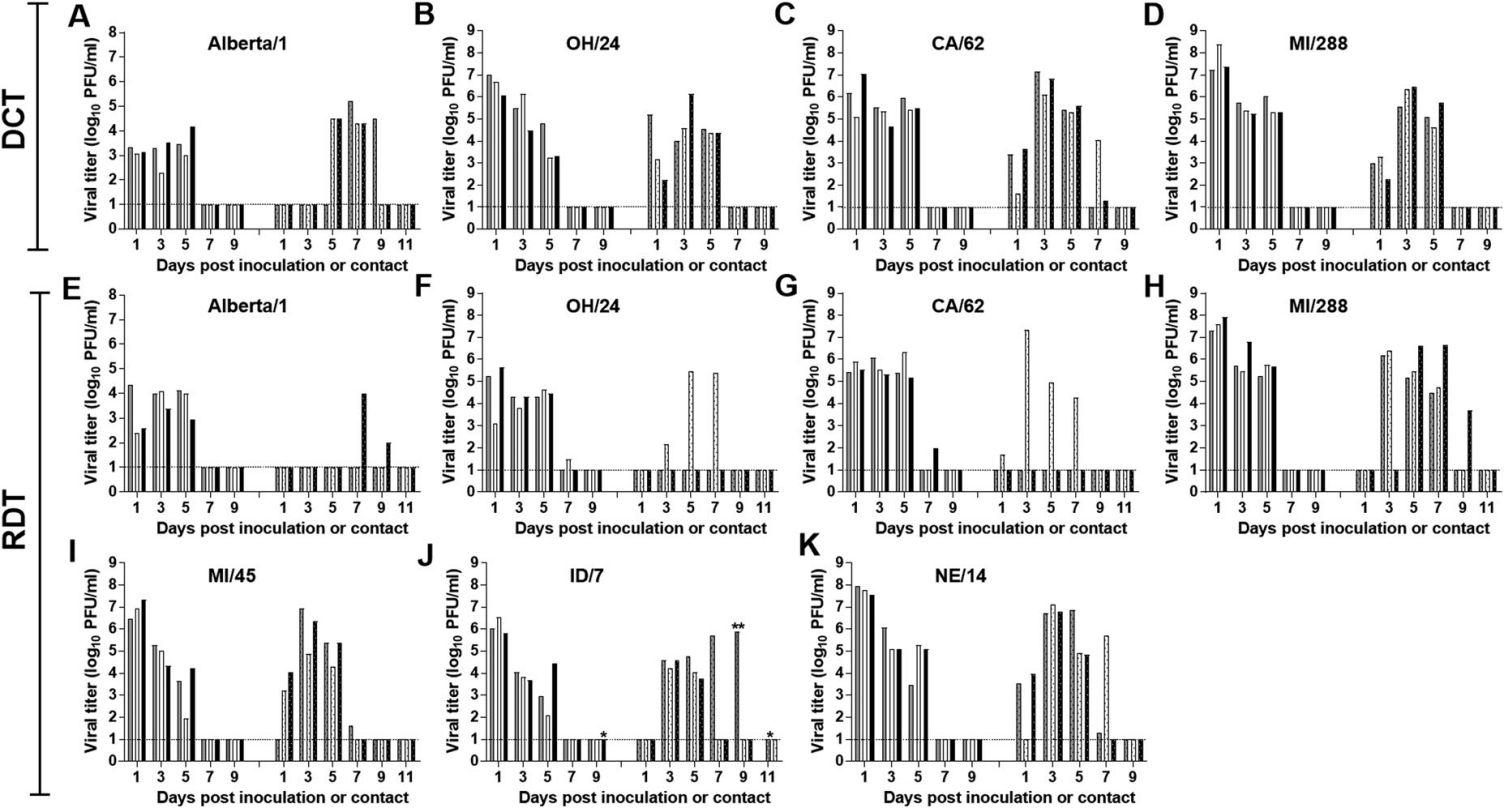
Transmission of A(H1) influenza viruses in ferrets. Ferrets were inoculated with 6 log_10_ PFU of the indicated virus. After 24 h, a naïve ferret was added to each cage housing an inoculated ferret for the Direct Contact Transmission (DCT) model (A-D) or to each cage adjacent to an inoculated ferret for the Respiratory Droplet Transmission (RDT) model (E-K). Nasal wash samples were collected on the days indicated and titered using a standard plaque assay. Virus titers in nasal wash samples collected from individual inoculated ferrets are shown on the left side of each panel while those from individual contact ferrets are shown on the right side of each panel. The limit of detection was 1 log_10_ PFU/ml (dashed line). *Ferrets were euthanized on day 9 post inoculation or day 11 post contact., respectively due to severe weight loss or dyspnea. ** Nasal wash was collected 8 days post contact., just prior to euthanasia.

Both DCT and RDT models were used to asses transmissibility of A(H1)v viruses, while the human adapted viruses, which are known to transmit between ferrets efficiently, were only tested using the RDT model to reduce the overall number of animals used in this study. Each of the A(H1)v viruses rapidly transmitted between all co-housed ferret pairs within the first day of exposure. MI/288 virus was also capable of airborne transmission between all ferret pairs by 5 days p.c. The other three A(H1)v viruses, Alberta/1, OH/24 and CA/62, transmitted between 1 out of the 3 ferret pairs in the RDT model; the 2 uninfected ferrets from each group were confirmed to be seronegative against the inoculum virus at day 20–21 p.c. All of the human-adapted A(H1N1)pdm09 viruses were able to transmit efficiently through the air by 3 days p.c. Similar clinical signs and disease outcomes were observed in all ferrets that were infected after exposure to an inoculated animal (data not shown). For example, 2 of the ID/7 virus contact animals displayed severe weight loss (14.5 and 25%) accompanied by nasal discharge or dyspnea and required euthanasia on day 8 and 11 p.c. (Figure 2). The ferret transmission models used here were able to discern the differences in transmission capability of variant and human-adapted, seasonal IAVs.

## Discussion

Since the isolation of the first swine A(H1N1) influenza virus in 1930 [18], the classical swine lineage viruses have evolved via drift and reassortment with avian and human influenza viruses into distinct genetic and antigenic clades. With respect to the HA, which is a major antigenic determinant of influenza viruses, several H1 subtype lineages are maintained in North American swine such as 1A.1/alpha, 1A.2/beta, 1A.3.1/gamma, 1B.2.1/delta 2, and 1B.2.2/delta 1 [23–25]. Viruses of each of these lineages have been previously reported to cross the species barrier and infect humans, as such, these strains have been carefully monitored and assessed for their pandemic potential using the ferret model [20]. In this study, we characterized four swine-origin A(H1) IAVs that were most recently isolated from humans and three human-adapted A(H1N1)pdm09 seasonal viruses for comparison. Data generated during studies such as these are indispensable in pandemic risk assessments using tools like the Influenza Risk Assessment Tool (IRAT) [26].

The antigenic relatedness of a newly emerged influenza virus in comparison to viruses that are circulating in people as well as viruses that were used to produce CVVs and stockpiled pre-pandemic vaccines is a critical parameter used by the IRAT. A previous study suggested that population immunity to the highly transmissible in our study MI/288 A(H1N1)v virus (1A.3.3.2 lineage) is likely high, showing that immune sera obtained from children and adults vaccinated with seasonal influenza as well as ferrets infected with seasonal influenza virus effectively inhibited the MI/288 virus [15]. In contrast to what was previously observed for the MI/288 virus, our results show that lineage 1A.1.1 Alberta/1 and lineage 1B.2.1 CA/62 viruses had little to no HI reactivity with child or adult human post-vaccination sera, indicating that humans, especially those of younger age, lack immunity to these viruses. Ferret antisera raised to the North American OH/24 and the OH/24-like CVV (IDCDC-RG59), also failed to inhibit Alberta/1 in HI tests further demonstrating that these viruses have increased potential to infect and spread among an immunologically naïve population and highlight the need for the development of new CVVs.

Beyond the antigenic and genetic differences displayed by swine-origin IAVs, the scope of disease progression and the ability to transmit can also substantially differ between strains. Similar to humans, ferrets are highly susceptible to influenza virus infection, display pronounced clinical symptoms and signs of infection, and can transmit virus to other ferrets [27]. For these reasons, the ferret represents the “gold standard” model for the study of the pathogenicity and transmissibility of newly emerging IAVs [28]. In published studies, human seasonal A(H1N1) viruses that circulated prior to the 2009 pandemic typically caused mild disease and were restricted to replication in the upper respiratory tract of ferrets [19, 21, 22]. In contrast, pandemic A(H1N1) viruses (from 1918 and 2009 pandemics) [9, 22, 29, 30], as well as previously tested swine-origin A(H1) subtype viruses [9, 31, 32] were detected throughout the respiratory tract of ferrets and less frequently at extrapulmonary sites such as olfactory bulb and intestines [31], progressing in some cases to severe disease and lethal outcome. The variant IAVs tested here caused transient signs of disease and virus spread was limited to respiratory tract tissues of all ferrets, with an exception. MI/288 virus was also found in the olfactory bulb of all inoculated animals. The human-adapted influenza viruses studied here displayed phenotypes similar to those reported for pre-2009 pandemic A(H1N1) IAVs [19, 21, 22]. MI/45 and ID/7 viruses were predominately detected in the upper respiratory tract. However, NE/14 virus displayed the most pronounced virus spread and was detected throughout the respiratory tract as well as in olfactory bulb and brain tissues, which is consistent with some 2009 pandemic isolates [22, 33]. While most ferrets inoculated with variant or human-adapted viruses typically recover from infection, the ID/7 A(H1N1)pdm09 strain displayed enhanced pathogenicity in the ferret model; one inoculated ferret and two contact ferrets succumbed to the infection due to excessive weight loss and/or severe signs of infection including diarrhea and dyspnea. Although uncommon, mortality was previously reported for some A(H1N1)pdm09 strains isolated in 2009 [22].

Unlike human, seasonal influenza viruses, which transmit efficiently between ferrets through the air, including the A(H1N1)pdm09 viruses used in this study, transmissibility of A(H1)v viruses varies between strains [20]. Barman et al. found that swine triple reassortant A(H1) viruses containing human-origin HA and NA genes transmitted efficiently between ferrets via the air, while viruses containing swine-origin or mixed-origin HA and NA genes displayed reduced transmissibility [34]. Consistent with these observations, representative triple reassortant viruses containing swine-like HA and NA genes, did not transmit through the air [32]. Interestingly, while the HA and NA gene lineage of triple reassortant swine viruses isolated prior the 2009 pandemic was somewhat predictive of virus transmission in the ferret model, the transmissibility profiles of A(H1)v viruses isolated post pandemic differed between strains and did not appear to be clade-dependent [20]. In the current study, one variant virus, MI/288 A(H1N1)v, transmitted as efficiently as the human, seasonal viruses, The enhanced transmission of this virus potentially could be due to the presence of HA, NA, and M genes of A(H1N1)pdm09 origin and the high titers of virus shedding detected in nasal washes of inoculated animals indicating progressing adaptation of this virus [35, 36]. Interestingly, OH/24 A(H1N2)v virus, which transmitted between a single ferret pair using the RDT model, possesses the same gene constellation as the previously studied A/MN/45/2016 A(H1N2)v virus, which transmitted via air between ferrets with 100% efficiency [31]. Both viruses share 97.3% amino acid identity in the NA gene and 99.1% amino acid identity in the remaining genes, showing that the transmission phenotypes can be modulated at the amino acid sequence level and are not necessarily dependent on more extensive genotypic distinctions.

Collectively, these data highlight the continued adaptation of A(H1N1) IAVs derived from the 2009 pandemic to humans and illustrate that, while infrequent, selected viruses nonetheless possess the ability to cause severe and fatal disease in mammals. Furthermore, while A(H1N1)v and A(H1N2)v viruses continue to sporadically cause human infections, their distinct antigenic profiles and the capacity for airborne transmission underscore the pandemic risk posed by these viruses. Continued assessment of antigenicity, pathogenesis, and transmission of novel and emerging influenza viruses from zoonotic reservoirs using the ferret model is necessary for pandemic preparedness.

## Supplementary Material

Supplemental Material
