# Supplementary figures and images for: Pathogenesis and transmission of human seasonal and swine-origin A(H1) influenza viruses in the ferret model
Source: Emerg Microbes Infect. 2022 Jun 1;11(1):1452–9. doi: 10.1080/22221751.2022.2076615 (PMC9176692; doi:10.1080/22221751.2022.2076615)

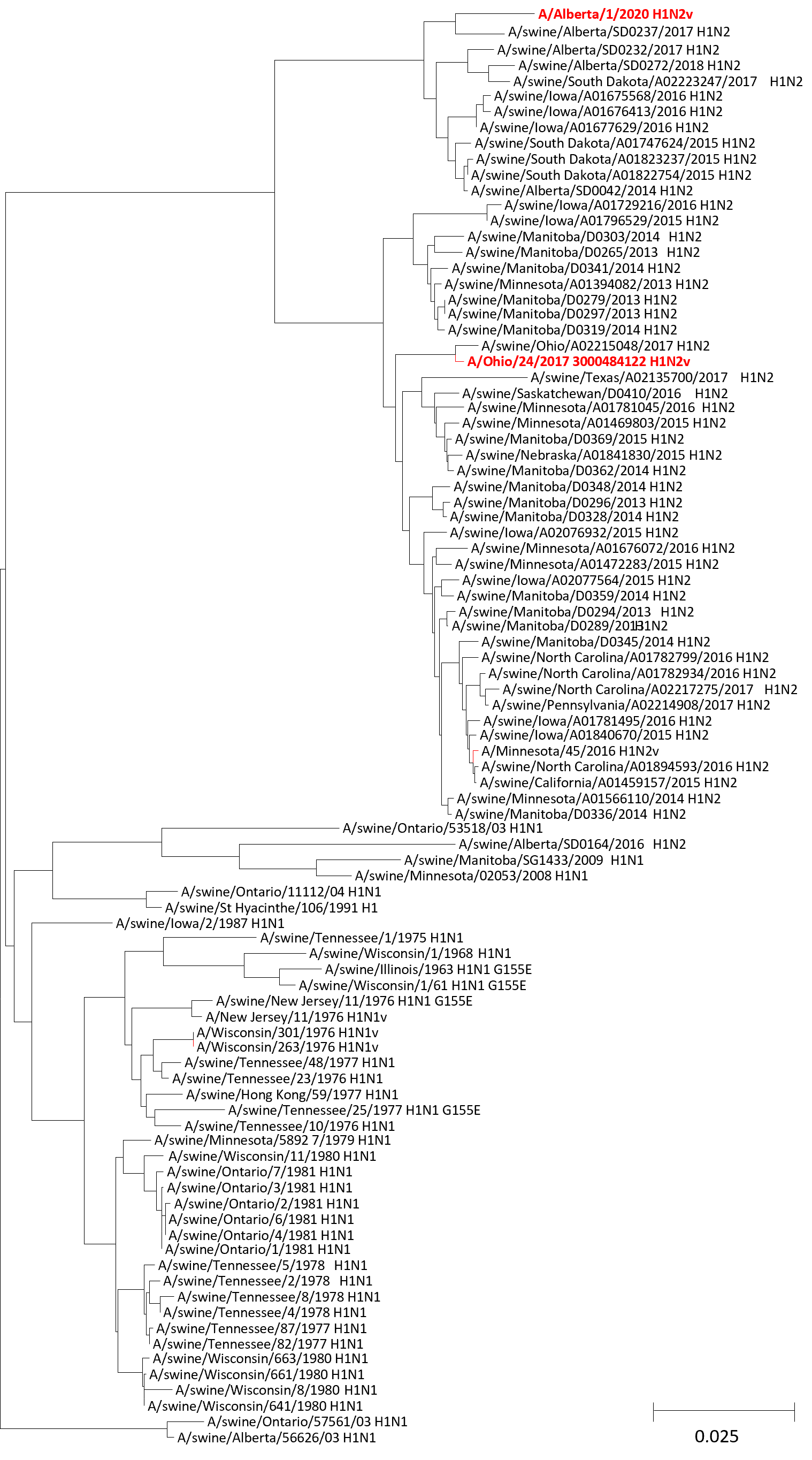

Supplement: Supplemental Material [file TEMI_A_2076615_SM0756.zip › Supplemental files/Supp Fig 1a.tif]

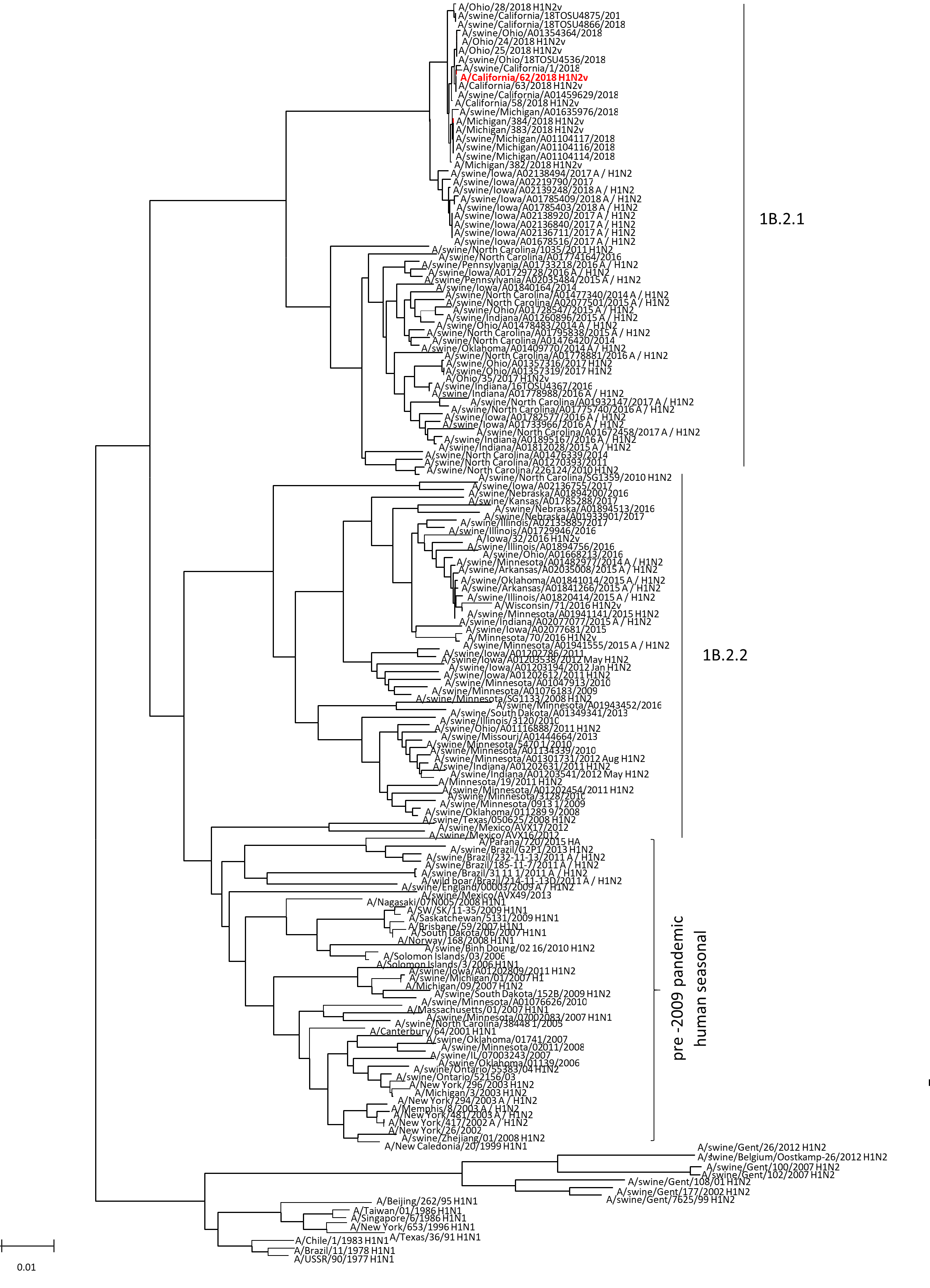

Supplement: Supplemental Material [file TEMI_A_2076615_SM0756.zip › Supplemental files/Supp Fig 1b.tif]
